# Anthelmintic Potential of Medicinal Plants against *Ancylostoma caninum*

**DOI:** 10.1155/2021/3879099

**Published:** 2021-11-28

**Authors:** Savira Ekawardhani, Utari T. Anggoro, Ita Krissanti

**Affiliations:** ^1^Parasitology Division, Department of Biomedical Sciences, Faculty of Medicine, Padjadjaran University, Sumedang, Indonesia; ^2^Veterinary Medicine Program, Faculty of Medicine, Padjadjaran University, Sumedang, Indonesia; ^3^Microbiology Division, Department of Biomedical Science, Faculty of Medicine, Padjadjaran University, Sumedang, Indonesia

## Abstract

*Ancylostoma caninum* is one of the most important hookworms in dogs. A study revealed that the prevalence of ancylostomiasis in Indonesia is relatively high. However, cases of persistent ancylostomiasis in dogs were reported, indicating the possibility of anthelmintic resistance. The aim of this review is to provide an overview of the anthelmintic potential of plants preclinically against *A. caninum* based on related research articles. This review retrieved 14 articles from 2001 to 2021 investigating 19 different plants. *Momordica charantia*, *Diospyros anisandra*, and *Citrus aurantiifolia* hold a promising prospect as anthelmintic against *A. caninum*. This review found aspects of those medicinal plants that need to be investigated deeper to improve our understanding of the matter. *In vitro* results in this review have not yet been tested in *in vivo* trials, which are essential in determining the efficacy and safety of the use of these medicinal plants and also to justify its clinical application.

## 1. Introduction

*Ancylostoma caninum* is one of the most important hookworms in dogs. A study revealed that the prevalence of ancylostomiasis in Indonesia is relatively high (92, 5% in West Java, 92, 31% in Yogyakarta, 88, 64% in Central Java, and 34% in the tourist area of Bali) [1]. Synthetic anthelmintics have been the most common and effective way to counter hookworm infection. For an anthelmintic of 90–100% efficacy, pyrantel is an approved anthelmintic used against *A. caninum* both as treatment and prevention. However, cases of recurrent or persistent ancylostomiasis in dogs that have been treated were reported. There could be possibilities leading to anthelmintic resistance, where hookworms have developed immunity to the anthelmintics used.

Anthelmintic resistance in dogs, especially greyhounds, might first come into existence from the breeding farms. Deworming protocols in the industry involve frequent use of synthetic anthelmintics, which might have contributed to the development of anthelmintic resistance. As stated by Jackson [2] and Kopp et al. [2] in their respective studies which sequentially used samples of Brisbane greyhounds and dogs of various breeds, the efficacy of pyrantel in both studies was revealed to have decreased from >90% to 75.1% and 25.7%, respectively. In 2019, another persistent case of ancylostomiasis caused by *A. caninum* was reported in greyhounds, miniature schnauzers, and mix hounds from Florida and Georgia, USA. According to Castro et al. [3], drugs that have been used in each case of treatment failure and discussed include pyrantel, praziquantel, fenbendazole, ivermectin, moxidectin, imidacloprid, febantel, mebendazole, and oxantel. These findings imply that resistance to several types of anthelmintics in *A. caninum* has been developed and may have spread to the general dog population, not limited to the greyhound in Brisbane, Australia. *A. caninum* is a zoonotic worm, which means it can attack humans through skin penetration by infective worm larvae. The development of anthelmintic resistance in these zoonotic worms will impact the number of drug options given to humans, which will become increasingly limited.

Not only in Australia, where greyhounds are bred as racing dogs, the trade of purebred dogs bred in Indonesia is also booming and found in many areas in Indonesia. To breed dogs of the best quality, namely, *breeders* or dog breeders will provide maximum care for their dogs, starting from adequate feed, vaccination, and anthelmintics as a prophylactic measure. Resistance to various types of anthelmintics in greyhound kennels in Australia is due to its intensive deworming protocol. Thus, it does not rule out the chance that the same phenomenon will also occur in Indonesia, where the deworming protocols are just as intensive. In the long term, apart from being a zoonotic threat, it will also be an economic loss. Medicinal plants can be an option for further research. The use of medicinal plants in veterinary medicine has been practiced for a while due to the variable availability and affordability of synthetic anthelmintics, especially in rural areas. This study aims to provide an overview of the anthelmintic potential of plants preclinically against *A. caninum* based on related research articles.

## 2. Methods

### 2.1. Selection Criteria

All references were screened for relevance by studying the title and abstract of each citation. A set of exclusion criteria were identified, which were as follows: (1) the literature was published in a language other than Indonesian and English, (2) the literature was an article review, (3) the literature discussed medicinal plants from a perspective other than anthelmintic, and (4) the article was related to herbal anthelmintic, but the subject used was not defined as *A. caninum*.

In the following step, the abstracts of the references were judged for exclusion and inclusion. A reference was finally included if (1) the article discussed the anthelmintic potential of medicinal plants, (2) the article discussed *A. caninum* as the subject of study, and (3) the article was a research article.

### 2.2. Search Strategy

A computerized search was performed using two databases: Google Scholar and Pubmed. To collect all relevant studies that were published in the range of 2001–2021, primary keywords, namely, medicinal plant, anthelmintic, and *Ancylostoma caninum,* were included along with other potential keywords.

### 2.3. Data Extraction

The references were analyzed for which parts of plant were being used, the chemical compound was suspected of anthelmintic properties, the extraction method was applied, the doses were used, the experimental models were applied (*in vitro* or *in vivo*), and the mechanism of action the plants was possessed as anthelmintic.

## 3. Results

### 3.1. Search Results

**Figure 1:**
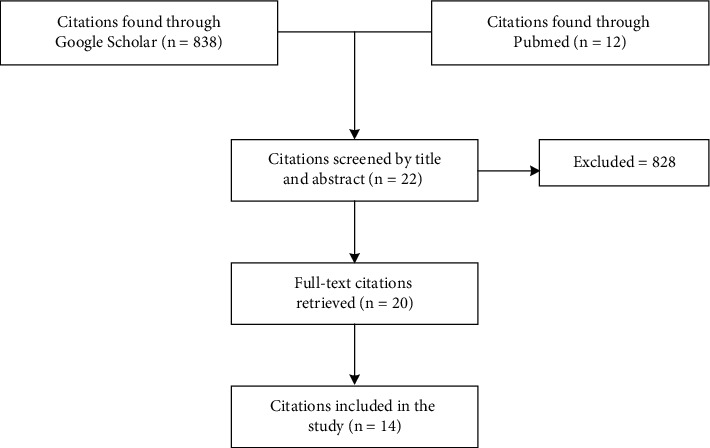
Study eligibility flowchart.

The search from databases resulted in the identification of 850 references in total. After initial screening, 22 of the 850 references remained. After references were excluded based on the applied criteria, only 14 references fulfilled the inclusion criteria.  Figure 1 depicts the article eligibility flowchart.

### 3.2. Characteristics of Included Studies

From the 14 included references, 8 studies were *in vitro* (Table 1) and 6 studies were *in vivo* (Table 2).

## 4. Discussion

### 4.1. In Vitro Assay

Out of the 14 plants tested *in vitro*, 12 plants (85.71%) showed an anthelmintic effect on eggs, larvae, and adult worms, while two other plants (14.28%) did not show an anthelmintic effect at the concentrations tested. EC50 and EC90 are the concentration of a substance that can show a characteristic effect on 50% and 90% of the biological subject material. LC100 and LC50 are the lowest concentration of a substance in defined medium that, under defined conditions, are lethal to 100% or 50% exposed organism, respectively. While IC50 is the concentration of drug required for 50% inhibition, dependent of the assay condition. They all determine the efficacy of medicinal plant material alongside other measures [4]. However, the data included in the references vary, both in terms of parameters and data presentation. Orengo et al. [5], in their research on the anthelmintic activity of *Allium cepa*, *Allium sativum*, and *Jatropha curcas*, said that *Allium cepa* and *Allium sativum* had LC100 at 156 g/mL and >156 g/mL, respectively, without concluding LC50 and IC50.

Different solvents can give different results. Orengo et al. [5] used ethanol extract and aqueous extract; in aqueous extract, the concentration required to obtain the same results would be greater than that of ethanol extract. Pone et al. [6] tested *Canthium mannii* with ethanol, cold water, and hot water and also found that the ethanol plant extract showed higher anthelmintic activity in egg hatching inhibition than that of cold water and hot water. However, this does not mean that researching the anthelmintic activity of medicinal plants using ethanol as a solvent is the right thing to do. According to Chagas [7], the choice of solvent is crucial to be considered in *in vitro tests* to avoid solvent incompatibility with worms, as it can give false positive results where the observed changes are interpreted as the pharmacological properties of medicinal plants.

Results may vary depending on which plant part is in tests and the time of plant collection. For example, the bark extract of *Diospyros anisandra* collected in the rainy season presents egg hatchability inhibition up to >95% at 75 g/mL. On the contrary, the bark extract of *Diospyros anisandra collected* in the dry season required at least 300 g/mL for hatchability inhibition of >94%. The leaf extract of *Diospyros anisandra collected* in the rainy and dry seasons on eggs *A. caninum* showed the lowest inhibition (25.9% and 5.2%, respectively) at 600 g/mL [8].

Anthelmintic effects of bioactive compounds in plants may vary depending on the stage of development of the worm. Two studies of *Mikania glomerata* using eggs and larvae revealed that the ethanol extract of *Mikania glomerata* at a concentration of 0.1–10 mg/mL could inhibit egg hatchability, while the ethanol extract of *Mikania glomerata* required at least 25 mg/mL and 50 mg/mL to reduce the number of larvae to 13.30% and 61.66%, respectively [9, 10].

*Thymus vulgaris*, *Parthenium hysterophorus*, *Passiflora laurifolia*, and *Momordica charantia* are plants recommended by *Vodou*, the priest in Haiti, to treat infections by worms *A. caninum*. Wolpert et al. [11] investigated the anthelmintic activity of these plants *in vitro* to observe the inhibition of larval feeding activity and to assess whether the inhibition was permanent. All *aqueous* extracts of these plants could inhibit larval feeding activity at various low concentrations, with 1.5 mg/mL being the lowest concentration and 3.5 mg/mL being the highest concentration. *Momordica charantia* can inhibit larval feeding activity at 1.5–2 mg/mL and is the only plant among the four plants where exposure causes irreversible inhibition of feeding activity in larvae, so it does not require continuous exposure. These results make *Momordica charantia* the most potent plant in the research of Wolpert et al. [11].

*Paullinia pinnata*'s hydroethanolic root extract was investigated against several worms: *A. caninum, Caenorhabditis elegans, Haemonchus contortus, Toxocara cati,* and *Trichuris vulpis*. The dominant compounds of *Paullinia pinnata, e*picatechin, and procyanidin, showed anthelmintic effects against *all* worm species tested except *A. caninum.* [12]

### 4.2. In Vivo Assay

There are six references (42.85%) that discuss the anthelmintic effect of 6 medicinal plants against *A. caninum in vivo*. Dogs (66.67%) and mice (33.34%) were used. The six medicinal plants were revealed to have anthelmintic properties by the fecal egg count (FEC). *Carica papaya* showed an anthelmintic effect of larval reduction up to 98.6% after 96 hours in mice infected with *A. caninum* [13]. Another plant, *Azadirachta indica,* reduced larval 159 count on mice after 72 hours [14]. Other plants tested on dogs, such as *Euphorbia hirta*, *Vernonia amygdalina, Allium cepa*, and *Citrus aurantiifolia*, also showed anthelmintic effects marked by varying reductions in the number of eggs obtained from fecal samples [15–18].

*A. caninum* is a blood-sucking worm that can cause anemia in infected dogs [19]. Hence, the anthelmintic activity in vivo was assessed by hematological parameters. The ethanol extract of *Citrus aurantiifolia* seeds was able to increase hemoglobin, erythrocyte, and hematocrit levels in the treated group, both in the group treated with only plant extract and in the group treated with a combination of plant extracts and mebendazole [17]. Similar results from articles discussing *Allium cepa*, *Euphorbia hirta*, and *Vernonia amygdalina* showed significant differences in hemoglobin, hematocrit, and erythrocyte levels compared to the control group [15, 18].

The inconsistency of the parameters studied in several articles was due to the absence of specific guidelines used in the anthelmintic potency of medicinal plants. The articles discussing *Carica papaya* and *Azadirachta indica* included hematological findings such as eosinophil level declined yet omitted information on erythrocyte, hematocrit, or hemoglobin levels [13, 14]. In this study, *Citrus aurantiifolia* was the only plant studied for its synergistic mechanism with mebendazole [17] and gave the best results among the treatment groups where the reduction in FEC reached 100%. Combination therapy has been widely used to achieve higher efficacy, as it targets different pathways of action. [17].

*Allium cepa* was the only plant in this review where both *in vitro* and *in vivo* data were obtained [5, 18]. The anthelmintic effect shown *in vivo* was not as good as that in *in vitro*, namely, reduction in the number of eggs only reached 47%, while hatchability of eggs reached 100% *in vitro*. The biotransformation processes in the GI tract of the animal could reduce the compounds *in vivo.*

### 4.3. Active Constituent

Terpenes are a group of plant chemical compounds putative of acting as anthelmintic in this study. Diterpenes of kaurane class compounds present in *Mikania glomerata* and *Mikania laevigata* showed anthelmintic activity in infective larvae of *A. caninum in vitro* [9, 10]. Low-level anthelmintic betulin and lupeol present in *Diospyros anisandra* are of triterpene compound [8]. Vernodalin and vernolide, present in Vern*onia amygdalina*, which can reduce the number of eggs *in vivo*, are sesquiterpene lactone compounds [15]. According to Adedapo [15], vernodalin and vernolide have an anthelmintic effect on the number of eggs with a mechanism that involves disruption in the mitochondrial ATP generation process. The aforementioned leads to decreased egg production by the worms in infected dogs due to exhaustion of energy. A similar mechanism is also assumed of the phenol compound C8H18O15 found in *Euphorbia hirta* [16].

Alkaloid pyridine, potassium quisqualata, and quisqualic acid are the putative anthelmintic substances present in the seeds of *Quisqualis indica*. Alkaloid pyridine and potassium quisqualata resemble synthetic anthelmintic, namely, levamisole and macrocyclic lactones, as they cause paralysis in worms [20]. Paralysis is a condition of impaired motor function due to lesions in muscles or nerves [1].

Papain, chymopapain, and lysozyme in *Carica papaya* are proteolytic enzymes [13]. The reduction in FEC obtained from this study is thought to be due to proteolytic enzymes that denature proteins in the worm cuticle. The cuticle is a component of the hydrostatic exoskeleton that regulates the locomotion of the worm. Damage to the cuticle will make the worm incapable of movement and suffer a slow death [21].

Quercetin is a polyphenolic compound present in *Euterpe edulis* [10]. Quercetin is known to have anti-inflammatory, antihypertensive, vasodilator, antiobesity, antihypercholesterolemic, and antiatherosclerotic activities [22, 23]. The article did not report the anthelmintic mechanism of quercetin.

## 5. Conclusion

Medicinal plants, especially *Momordica charantia*, *Diospyros anisandra*, and *Citrus aurantiifolia*, hold a promising prospect as anthelmintic against *A. caninum*. However, there are still many aspects of those medicinal plants that need to be investigated deeper to improve our understanding of the matter. *In vitro* results in this review have not yet been tested in *in vivo* trials, which are essential in determining the efficacy and safety of the use of these medicinal plants, also to justify its clinical application.

## Figures and Tables

**Table 1:** *In vitro* studies on the effects of plants against *Ancylostoma caninum*.

Author	Country	Plant	Plant part used	Extract	Results
Burgos-Flota et al.	Mexico	*Diospyros Anisandra*	L, bark	Methanolic	LC_50_ = 60 *μ*g/mL
					LC_99_ = 76.7 *μ*g/mL
Orengo et al.	Kenya	*Allium sativum*	Bulb	Ethanolic	LC_100_ ≥ 156 *μ*g/mL
					IC_100_ = 10000–5000 *μ*g/mL
Orengo et al.	Kenya	*Allium cepa*	Bulb	Ethanolic	LC_100_ = 156 *μ*g/mL
					IC_100_ = 10000–2500 *μ*g/mL
Orengo et al.	Kenya	*Jatropha curcas*	Leaf	Aqueous	No anthelmintic activity at 156.25–10000 g/mL against eggs and larvae
Tonini et al.	Brazil	*Euterpe edulis*	N/A	Ethanolic	EHI = 0.1 mg/mL; 1 mg/mL; 10 mg/mL
Araújo et al.	Brazil	*Mikania glomerata*	N/A	Ethanolic	Reduction in the number of L3 at 25 mg/mL and 50 mg/mL was 13.30% and 61.66%, respectively
Tonini et al.	Brazil	*Mikania Glomerata*	N/A	Ethanolic	EHI = 0.1 mg/mL; 1 mg/mL; 10 mg/mL
Tonini et al.	Brazil	*Mikania laevigata*	N/A	Ethanolic	EHI = 0.1 mg/mL; 1 mg/mL; 10 mg/mL
Pone et al.	Cameroon	*Canthium mannii*	Bark	Ethanolic	LC_90_ = 1000 *μ*g/mL
Mulyaningsih	Indonesia	*Quiqualis indica*	Seed	Aqueous	LC_50_ = 25.78%
Wolpert et al.	USA	*Thymus vulgaris*	N/A	Aqueous	Inhibits larval feeding 100% at 2.5–3 mg/mL
Wolpert et al.	USA	*Parthenium hysterophorus*	N/A	Aqueous	Inhibits larval feeding 100% at 2–2.5 mg/mL
Wolpert et al.	USA	*Passiflora laurifolia*	N/A	Aqueous	Inhibits larval feeding 100% at 3.5 mg/mL
Wolpert et al.	USA	*Momordica charantia*	N/A	Aqueous	Inhibits larval feeding 100% at 1.5–2 mg/mL
Spiegler et al.	Germany	*Paullinia pinnata*	Root	Hydroethanolic	No anthelmintic activity against A. caninum larvae

N/A, not available.

**Table 2:** *In vivo* studies on the effects of plants against *Ancylostoma caninum*.

Author	Country	Plant	Plant part used	Animal model	Treatment	Results
Bi & Goyal	India	*Carica papaya*	N/A	Mouse	0.2 mL 14 an 7 days before infection with 500 larvae	Larval reduction 98.6% after 96 hours
Hassanain et al.	Egypt	*Citrus aurantiifolia*	Seed	Dog	Combination of plant extract 40 mg/kg mebendazole 50 mg/kg per day for two weeks	FEC 100% reduction after two weeks
Bi & Goyal	India	*Neem/Azadirachta indica*	N/A	Mouse	0.2 mL 14 and 7 days before infection with 500 larvae	Larval reduction after 72 hours
Orengo et al.	Kenya	*Allium cepa*	Bulb	Dog	10 mL plant extract	FEC reduction 47%
Adedapo et al.	Nigeria	*Euphorbia hirta*	Leaf	Dog	500 mg/mL given three days in a row in 2 stages per 2 weeks IM and PO	FEC reduction 100% at the second stage
Adedapo et al.	Nigeria	*Vernonia amygdalina*	Leaf	Dog	500 mg/mL given consecutive days PO	FEC reduction 47.5% after two weeks

N/A, not available.

## Data Availability

This review includes the main part of the data generated or analyzed during the study. Other relevant data will be available from the corresponding author as necessary.
